# Detection of Virulence Genes in Multidrug Resistant Enterococci Isolated from Feedlots Dairy and Beef Cattle: Implications for Human Health and Food Safety

**DOI:** 10.1155/2019/5921840

**Published:** 2019-06-17

**Authors:** Frank Eric Tatsing Foka, Collins Njie Ateba

**Affiliations:** Department of Microbiology, School of Biological Sciences, Faculty of Natural and Agricultural Sciences, North-West University, Mafikeng, Private Bag X2046, Mmabatho, South Africa

## Abstract

The misuse/abuse of antibiotics in intensive animal rearing and communities led to the emergence of resistant isolates such as vancomycin-resistant enterococci (VREs) worldwide. This has become a major source of concern for the public health sector. The aim of this study was to report the antibiotic resistance profiles and to highlight the presence of virulence genes in VREs isolated from feedlots cattle of the North-West Province of South Africa. 384 faecal samples, 24 drinking troughs water, and 24 soil samples were collected aseptically from 6 registered feedlots. Biochemical and molecular methods were used to identify and categorise the enterococci isolates. Their antibiotic resistance profiles were assessed and genotypic methods were used to determine their antibiotic resistance and their virulence profiles. 527 presumptive isolates were recovered, out of which 289 isolates were confirmed as* Enterococcus *sp. Specifically,* E. faecalis* (9%),* E. faecium* (10%),* E. durans* (69%),* E. gallinarum* (6%),* E. casseliflavus* (2%),* E. mundtii *(2%), and* E. avium* (2%) were screened after molecular assays.* VanA *(62%),* vanB* (17%), and* vanC* (21%) resistance genes were detected in 176* Enterococcus* sp., respectively. Moreover,* tetK *(26),* tetL *(57),* msrA/B *(111), and* mefA *(9) efflux pump genes were detected in 138 VRE isolates. Multiple antibiotic resistances were confirmed in all the VRE isolates of this study; the most common antibiotic resistance phenotype was TET^R^-AMP^R^-AMX^R^-VAN^R^-PEN^R^-LIN^R^-ERY^R^.* CylA*,* hyl*,* esp*,* gelE*, and* asa1* virulence genes were detected in 86 VREs with the exception of vancomycin-resistant* E. mundtii* isolates that did not display any virulence factor. Most VRE isolates had more than one virulence genes but the most encountered virulence profile was* gelE-hyl*. Potentially pathogenic multidrug resistant VREs were detected in this study; this highlights the impact of extensive usage of antimicrobials in intensive animal rearing and its implications on public health cannot be undermined.

## 1. Introduction

Known as one of the main causes of nosocomial infections, enterococci are ubiquitous, gram positive, catalase negative, and facultative anaerobes that thrive as part of the normal microbiota in the gastrointestinal tract of humans and warm-blooded animals [1]. Used sometimes as probiotics, they may therefore be found in fermented food items but are very common in the soil and surface water and on plants and vegetables [1, 2]. Enterococci are opportunistic pathogens that cause diseases in immunocompromised patients as well as those who are admitted in intensive care units for long periods or those who have severe underlying sicknesses [3]. In fact, enterococci can induce bacteraemia, endocarditis, urinary tract infections, sepsis, burn wound, and deep tissue infections in such patients [4]. They can also cause intramammary infection and clinical mastitis in dairy cattle [5].

The emergence of multidrug-resistant strains such as vancomycin-resistant enterococci (VRE), as a result of extensive usage and misuse of antibiotics in intensive animal rearing and in clinical settings for the treatment of community-acquired infections, has become a major source of concern worldwide; the reason is the limited therapeutic options for the treatment of illnesses caused by such strains [6] and the ability of such strains to transfer genetic resistance determinants to other commensals of the gastrointestinal tract or to other bacterial strains in the environment [7]. VRE emerged as a result of the usage of avoparcin (a glycopeptide analogue of vancomycin) as a growth promoter in animal husbandry [8]. Consequently, it was banned worldwide due to the possible transmission of VRE from farm animals to humans. Growth promoters are believed to ameliorate feed conversion and animal growth and reduce mortality and morbidity rates resulting from clinical and subclinical illnesses, although the mechanism through which this is achieved is still poorly understood [9]. VREs were isolated for the first time in the late 1980s [10]. Since then, there have been several reports of VRE detection worldwide. In fact, VREs have been detected in food animals, in retail meat, in vegetables, in drinking water, and in underground and surface water as well as being among hospitalised and nonhospitalised people [11–16]. The continuous isolation of VREs worldwide suggests that avoparcin may not be the absolute factor of the spread and the dissemination of VRE in animals and in the environment, probably because co-selection of resistance genes located on the same mobile genetic elements does occur as a result of the usage of other antibiotics in animal rearing [11–16]. Resistance to vancomycin is either intrinsic or acquired through the possession of eight types of vancomycin resistance genes (*vanA*,* vanB*,* vanC*,* vanD*,* vanE*,* vanG*,* vanL*,* vanM*, and* vanN*) [7].

Two groups of tetracycline resistance genes have been reported so far. The first group comprises* tetM*,* tetO*, and* tetS* resistance genes which confer resistance through ribosomal protection; meanwhile the second group encompasses* msrA/B*,* mefA*,* tetK*, and* tetL* genes which confer resistance through efflux pump mechanisms. Although a* tetU* resistance gene has been reported to trigger low level resistance, the mechanism through which this is achieved is still unknown [17].

The outstanding ability of enterococci to cause illnesses is due to the possession of virulence factors, some of which include* gelE*,* esp*,* cylR*_*1*_,* cylR*_*2*_,* cylL*_*l*_,* cylLs*,* cylM*,* cylB*,* cylA*,* cylI*,* hyl, *and* asa1* [18]. Virulence factors are yet to be characterised extensively and seem to differ among enterococcal species. So far, it has been demonstrated that the enterococcal surface protein* esp* promotes the colonization of host cells while* hyl* and* gelE* genes promote the production of toxic substances which have a destructive effect on the host's tissues.* Cyl* genes promote the production of cytolysin, a protein which enables pathogenic enterococci to escape the host immune system by destroying macrophages and neutrophils. Moreover,* asa1* mediates the production of aggregation substances [18].

Antimicrobials are routinely used in animal husbandry but there are few surveys determining the contribution of intensive cattle rearing in the dissemination of multidrug resistant VREs into the different ecological niches. The use of antimicrobials in intensive animal rearing has been identified as one of the main causes of multidrug resistant strains emergence [19]. The ability of enterococci to acquire antibiotic resistance through diverse mechanisms (plasmids and transposons, chromosomal exchange, or mutation) is quite challenging as far as therapeutic options are concerned [6]. Therefore, a constant genotypic monitoring of the different resistant enterococcal isolates is vital for food industry and public health institutions as well as environment protection agencies.

This study is part of a larger project which assesses the contribution of cattle feedlots to the dissemination and the spread of VREs in the environment. Its aims were to screen and characterize VRE isolates from dairy and beef cattle feedlots and to identify characteristics of public health importance to which humans could be exposed (directly or indirectly) such as their antibiotic resistance and their virulence profiles.

## 2. Materials and Methods

Ethical clearance was obtained from the North West University ethics committee before sample collection.

### 2.1. Sample Collection and Area of Study

Collection of samples was performed in six registered commercial feedlots located in the North-West province of South Africa. The samples were collected depending on the availability of the cows in the kraals and the permission from the feedlots owners. Faecal samples from dairy and beef cattle were collected aseptically by rectal palpation with sterile armed-length gloves, preserved in Cary-Blair medium, and transported on ice to the lab for processing with aseptically collected samples of water from the drinking troughs and soil samples from the kraals (Table 1).

### 2.2. Isolation of VRE from the Samples

Faecal samples were 10-fold serially diluted with buffered peptone water. Inoculation of the diluted faecal slurries was made onto bile-esculin azide agar (Biolab, South Africa) supplemented with 6*μ*g/ml vancomycin (Sigma-Aldrich, Johannesburg, South Africa). An aliquot of 100 ml from each water sample was filtered through a 0.45 *μ*m (47mm grid) sterile filter membrane (PALL Life Sciences, Mexico) on a vacuum water pump machine (Model: Sartorius 16824); the membrane filters were placed with a sterile forceps onto bile-esculin azide agar (BEA) (Biolab, South Africa) supplemented with vancomycin (6 *μ*g/ml) to select for VRE. The soil samples were put in buffered peptone water and submitted to a cell disruptor (model N° SI-D257, Scientific Industries Inc., USA). The supernatant was 10-fold serially diluted and inoculated onto bile-esculin azide agar (BEA) (Biolab, South Africa) supplemented with vancomycin (6 *μ*g/ml). After 24-48h incubation at 37°C, brown to black colonies from the plates were isolated and tested for catalase and Gram staining. Gram positive and catalase negative isolates were streaked in order to harvest pure colonies that were kept as stock culture at -80°C into Luria-Bertani broth supplemented with 50% glycerol after 24h incubation, for further characterization.

### 2.3. Genomic Enterococcus DNA Isolation and Identification

Pure colonies were revived onto nutrient agar, cultured overnight at 37°C in 20 ml brain heart infusion broth (BHI, Merck, South Africa) and harvested through centrifugation. Genomic DNA was extracted with a DNA extraction kit (Zymo Research Genomic DNATM–Tissue MiniPrep Kit, ZR Corp., Irvine, USA) according to the manufacturer's instructions. The genomic DNA was quantified using a NanoDrop TM 1000 spectrophotometer (Thermo Fischer Scientific, USA). The 16S rRNA (Table 2) was amplified using oligonucleotide primer combinations and cycling conditions in Table 2 and a DNA thermal cycler (C1000 Touch™, BIO-RAD, California, USA). All the primers used in this study were sequenced by Inqaba Biotech (Pretoria, South Africa). PCR reactions were performed in 25 *μ*l standard volumes that comprised 12.5 *μ*l of 1X Master mix, 0.25 *μ*l of each 1*μ*M primer, 2*μ*l template DNA (20–30 ng/*μ*l), and 10 *μ*l nuclease free water. Sequence data were analysed using Geospiza FinchTV (version 1.4). All amplified DNA sequences were purified with a Zymo DNA Sequencing Clean-up Kit (Zymo Research Corp., Irvine, USA). The amplicons were sequenced by Inqaba Biotec (Pretoria, South Africa) and the raw sequence data were transferred on Geospiza FinchTV (version 1.4) to view the chromatograms. The sequences were identified using BLAST search on NCBİ web tools (http://www.ncbi.nlm.nih.gov/BLAST). Representative bacterial 16S rRNA sequences were submitted to the GenBank database under accession numbers MK086096- MK086108.

### 2.4. Species-Specific PCR Assay for the Identification of Enterococcus spp.

The identities of the different isolates were determined using previously described multiplex PCR assays designed to amplify the* ddl* gene specific to* Enterococcus faecalis* and* Enterococcus faecium* and species-specific superoxide dismutase (*sodA*) genes to* Enterococcus durans*,* Enterococcus gallinarum*,* Enterococcus hirae*,* Enterococcus casseliflavus*,* Enterococcus mundtii*, and* Enterococcus avium* [21, 24]. Amplifications were performed using a DNA thermal cycler and volumes described in the previous paragraph. The primers sequences and their cycling conditions appear in Table 2.* E. faecalis *strain ATCC 29212 was used as the positive control strain while* Staphylococcus aureus* ATCC 43322 was used as the negative control.

### 2.5. PCR Detection of Vancomycin Resistance, Tetracycline Efflux Pump, and Virulence Genes

The presence of vancomycin resistance determinants (*vanA*,* vanB*, and v*anC*) in the enterococcal strains was assessed using a multiplex PCR analysis with specific primers and PCR conditions previously described by Depardieu* et al.* [21]. The final 20*μ*l volume contained 1 *μ*l genomic DNA sample, 12.5*μ*l DreamTaq PCR Master Mix, 0.5*μ*l of each 1*μ*M primer, and 6 *μ*l nuclease-free water.* E. faecium* BM4147 (*vanA*),* E. faecalis* NCTC 13379 (*vanB*), and* E. gallinarum* BM4174 (*vanC*) were used as positive control strains while* Staphylococcus aureus* ATCC 43322 was used as the negative control.

Resistance to tetracycline was assessed through the amplification of tetracycline resistance genes, precisely* msrA/B*,* mefA*,* tetK, tetM*, and* tetL* genes as described in previous studies [13, 17]. The final 25*μ*l volumes contained 1 *μ*l genomic DNA sample, 12.5 *μ*l DreamTaq PCR Master Mix, 0.5 *μ*l of each 1*μ*M primer, and 10,5 *μ*l nuclease-free water.

The virulence determinants of VRE isolates were determined through the amplification of the* asa1, cylA, esp, gelE, *and* hyl *gene sequences using chromosomal DNA extracted from the isolates [13]. Final volumes of 25 *μ*l contained 70ng/*μ*l of genomic DNA, 0.2*μ*M of primers* asa1* and* gelE* each, and 0.4*μ*M of primers* cylA*,* esp*, and* hyl* each. The primers sequences and the cycling conditions appear in Table 2.

### 2.6. Gel Electrophoresis of the Amplicons

Amplicons were separated by electrophoresis on a 1.5% (w/v) agarose gel (containing 0.001*μ*g/ml ethidium bromide) using 1 X TAE (40 mM Tris (pH 7.6), 20 mM acetic acid, and 1 mM EDTA) at 80V for 15 minutes and later on at 60V for 4 hours. A ChemiDoc Imaging System (BIO-RAD ChemiDoc™ MP Imaging System, Hercules, California, USA) was used to capture the image using Gene Snap (version 6.00.22) software. Each gel contained a 100 bp or 1 kb molecular weight marker (BioLab, New England).

### 2.7. Antimicrobial Susceptibility Test

The VRE isolates were tested against nine antibiotics (Mast Diagnostics, UK) using the Kirby-Bauer disc diffusion method [24]. This assay was performed on Mueller Hinton agar (Merck, South Africa) using tetracycline (TET 30 *μ*g), ampicillin (AMP 10 *μ*g), amoxicillin (AMX 10 *μ*g), vancomycin (VAN 30 *μ*g), chloramphenicol (CHL 30 *μ*g), penicillin (PEN 10 *μ*g), linezolid (LIN 30 *μ*g), ciprofloxacin (CIP 5*μ*g), and erythromycin (ERY 15*μ*g).* Staphylococcus aureus* ATCC 43322 was used as control strain and the zones of inhibition were determined using the Clinical and Laboratory Standards Institute (CLSI) guide (2013). The isolates were grouped as resistant or multidrug resistant based on the occurrence of resistance to one or more antimicrobials. MIC test was also carried out on the VRE isolates using vancomycin and linezolid MIC strips (Liofilchem s.r.l., Via Scozia, Italy) as per the manufacturer's protocol and the results were recorded.

### 2.8. Data Analysis

RStudio package (version 3.5) and Statistica 13 (StatSoft, TIBCO software Inc., USA) were used to organise, analyse, and compute the data derived from this study. The proportions were used to describe the observations of the different characteristics for which the isolates were screened.

## 3. Results

### 3.1. Species Distribution and Occurrence of Vancomycin-Resistant Enterococci in the Feedlots and the Feedlots Cattle

384 faecal samples, 24 drinking troughs water, and 24 soil samples were collected from feedlots and feedlots cattle herds, making a total of 432 samples (Table 1). On the basis of biochemical and agar plate assays, 527 presumptive isolates were recovered, out of which 289 isolates were confirmed as* Enterococcus *sp. after molecular assays (Table 3).

**Figure 1:**
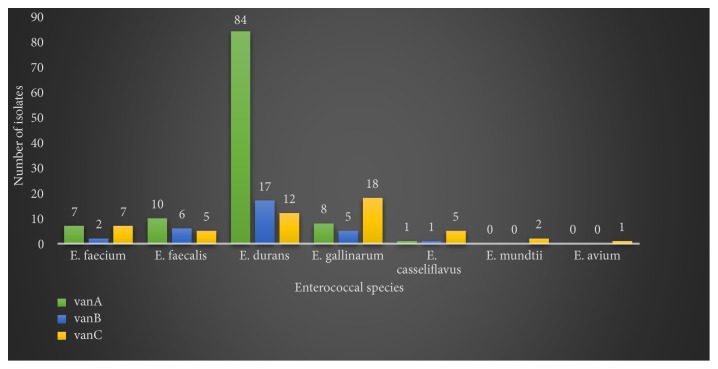
Vancomycin resistance genes trend in the enterococcal isolates from the feedlots.

Based on the species-specific PCR assays, the presumptive enterococcal isolates were identified as* E. faecalis* (9%),* E. faecium* (10%),* E. durans* (69%),* E. gallinarum* (6%),* E. casseliflavus* (2%),* E. mundtii *(2%), and* E. avium* (2%), while no* E. hirae* was isolated in this survey. 176 confirmed enterococcal isolates possessed vancomycin resistance genes. Precisely,* vanA*,* vanB*, and* vanC* resistance genes were detected in 110, 31, and 38 isolates, respectively (Figure 1), out of which 12, 6, and 5 isolates were screened from soil while 8, 2, and 1 isolates were screened from drinking trough water samples, respectively. Moreover, more than one vancomycin resistance gene was detected in some isolates.

**Figure 2:**
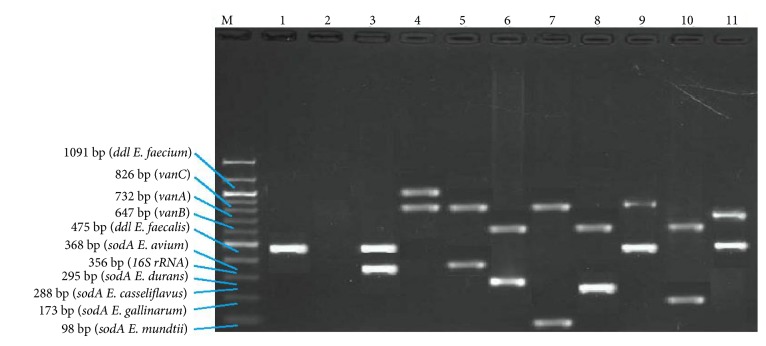
Multiplex PCR positive isolates. Lane M = marker 100-1000bp; lane 1 = positive control; lane 2 = negative control; lane 3 =* E. faecalis* and 16SrRNA; lane 4 =* vanC* positive* E. faecium*; lane 5 =* vanC* positive* E. avium*; lane 6 =* vanB* positive* E. durans*; lane 7 =* vanC* positive* E. mundtii*; lane 8 =* vanB* positive* E. casseliflavus*; lane 9 =* vanC* positive* E. faecalis*; lane 10 =* vanB* positive* E. gallinarum*; lane 11 =* vanA* positive* E. faecalis.*

Furthermore,* vanA* and* vanB* genes were mostly detected in* E. durans* isolates and absent in* E. mundtii *and* E. avium* isolates which possessed only* vanC* resistance gene (Figure 1). Representative amplicons of the VREs are displayed in Figure 2.

**Figure 3:**
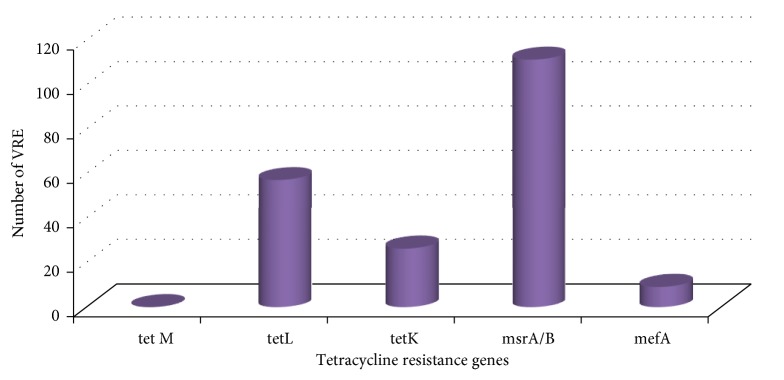
Distribution of tetracycline-resistant VREs.

138 (78%) VRE isolates possessed tetracycline resistance genes. Precisely, 26 (15%), 57 (32%), 111 (63%), and 9 (5%) VRE isolates possessed* tetK*,* tetL*,* msrA/B, *and* mefA* tetracycline efflux pump genes, respectively, while no* tetM* gene was detected amongst the VRE isolates (Figure 3).

**Figure 4:**
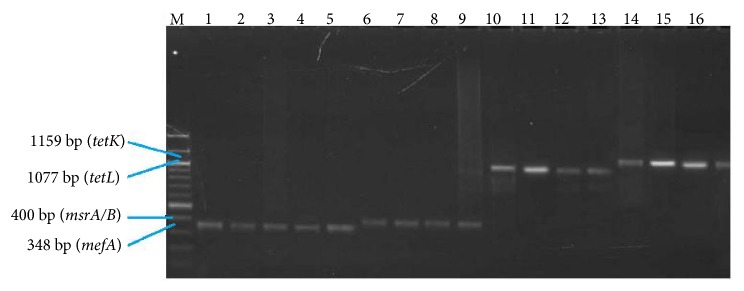
Lane M = 100 - 1000 bp marker; lanes 1 to 5 =* mefA* positive VREs; lanes 6 to 9 =* msrA/B* positive VREs; lanes 10 to 13 =* tetL* positive VREs; lanes 14 to 17 =* tetK* positive VREs.

56 (31%) VREs possessed more than one tetracycline resistance gene and the most encountered tetracycline resistance gene pattern encountered was* tetL-msrA/B*.  Figure 4 displays amplicons of tetracycline resistance genes after gel electrophoresis of VRE isolates.

### 3.2. Antibiotic Resistance Profile of the VRE Isolates

**Figure 5:**
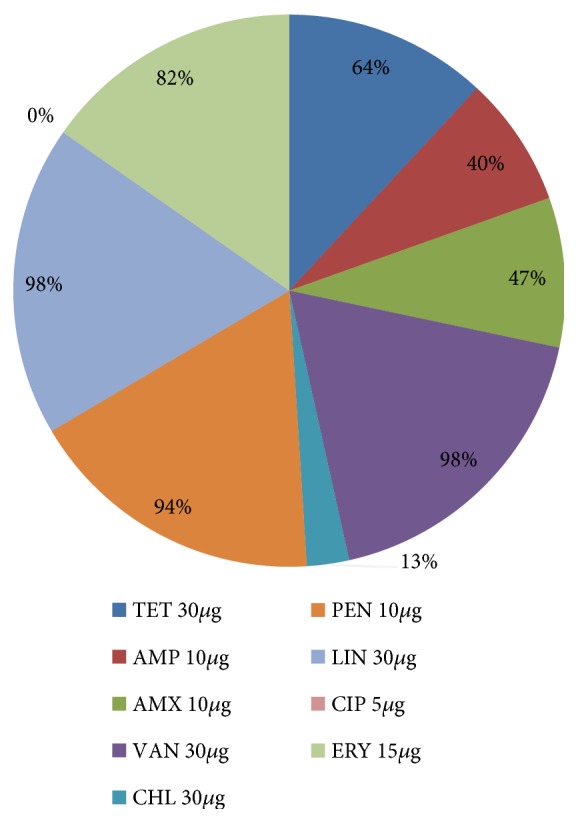
Proportions of antibiotic resistant VRE isolates.

Only vancomycin resistant enterococci were subjected to antimicrobial susceptibility assay and multidrug resistance was highly observed among the tested isolates (Figure 5). Almost all the isolates were resistant to vancomycin (98%) and linezolid (98%) as compared to ciprofloxacin which was effective on all the isolates (0% resistance). High resistance was also observed for penicillin (94%) and erythromycin (82%) while low resistance was observed with chloramphenicol (13%). 64%, 47%, and 40% of the isolates were resistant to tetracycline, amoxicillin, and ampicillin, respectively (Figure 5). The predominant multidrug resistance patterns that were observed among the isolates are displayed in Table 4.

The MIC test results were ranging from 192 to 256 *μ*g/ml for vancomycin and linezolid, meaning a high resistance of the VRE isolates to these two antimicrobials.

### 3.3. Virulence Profiles of the VRE Isolates

**Figure 6:**
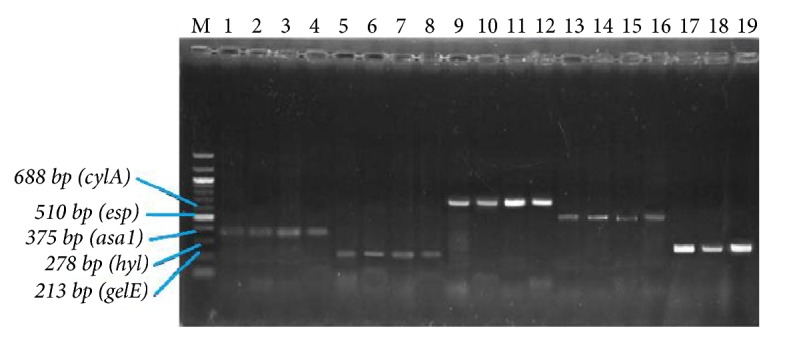
Enterococcal strains with virulence genes. Lane M = 100 – 1000 bp marker; lanes 1 to 4 =* asa1* positive VREs; lanes 5 to 8 =* gelE *positive VREs; lanes 9 to 12 =* cylA *positive VREs; lanes 13 to 16 =* esp *positive VREs; lanes 17 to 19 =* hyl* positive VREs.

Out of 176 VREs isolated, 86 (49%) VREs were found to possess virulence genes (Table 5). Some isolates exhibited multiple virulence genes but the most encountered virulence pattern was* gelE-hyl* (30 isolates) and this profile was mostly detected in vancomycin-resistant (VR)* E. durans* isolates. Moreover, virulence genes were detected in 8 (9%) VR* E. faecalis*, 8 (9%) VR* E. faecium*, 57 (67%) VR* E. durans*, 9 (11%) VR* E. gallinarum*, 3 VR (3%)* E. casseliflavus*, and 1 (1%) VR* E. avium* (Table 5). However, no virulence genes were detected in the vancomycin-resistant* E. mundtii* isolates. The only vancomycin-resistant* E. avium* isolated in this survey possessed only* gelE* virulence gene (Table 5).  Figure 6 displays amplicons of virulence genes after gel electrophoresis of virulent VRE isolates. All the VREs isolated from trough drinking water and soil samples possessed virulence genes.

### 3.4. Data Analysis

**Figure 7:**
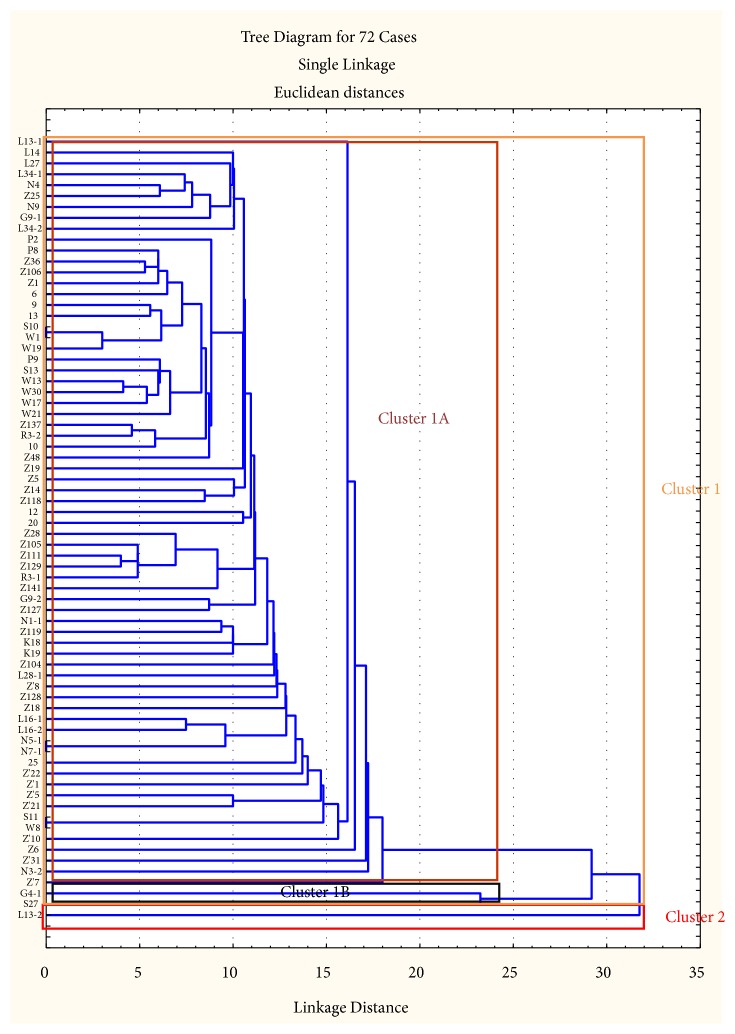
Dendrogram depicting the relationship between 72 multidrug resistant VREs isolated from the feedlots. Bacterial designations are based on sampling site and sample type.

72 VREs out of the 176 VREs isolated were clustered based on their inhibition zone diameters (Figure 7). The generated dendrogram was analysed and the results are shown in Table 6. Two major clusters were generated (clusters 1 and 2); cluster 1 had two subclusters (1A and 1B) while cluster 2 had only one isolate. Subcluster 1A was the largest cluster with 69 isolates while subcluster 1B had only 2 isolates. The only isolate in cluster 2 is of faecal origin; meanwhile the isolates in cluster 1 originated from faecal, soil, and water samples.

## 4. Discussion

Enterococci colonize the gut of mammals [3]. As they can thrive in any environment, they have been reported to be responsible for quite a number of life threatening conditions [4, 25]. The ability to cause infections in their hosts is due to the possession of virulence factors and antibiotic resistance genes which enable them to evade antimicrobial mechanisms of action [26, 27]. Genetic antimicrobial resistance attributes are either intrinsic or acquired and can be transmitted either among themselves or to other bacteria in the environment [27]. As far as vancomycin resistance is concerned, VanA, VanB, and VanC phenotypes are mediated by* vanA*,* vanB*, and* vanC* resistance gene clusters. VanA type of resistance is highly transferable to vancomycin and teicoplanin while VanB phenotypes are susceptible to teicoplanin. On the contrary, VanC resistance appears to be an intrinsic attribute of* E. gallinarum* and* E. casseliflavus* characterised by a low level of resistance to vancomycin [28]. Data of VREs from clinical settings or environmental sources are of utmost importance in epidemiological surveys for public health stakeholders, especially in countries where the prevalence of HIV-AIDS and diseases such as diabetes is high.

In this study, the prevalence of VREs in the feedlots and feedlots cattle was reported as well as their virulence and their antimicrobial susceptibility profiles. Out of 527 presumptive isolates, 289 (55%) bacteria were identified as enterococci, namely,* E. faecalis* (26),* E. faecium* (30),* E. durans* (199),* E. gallinarum* (18),* E. casseliflavus* (5),* E. mundtii* (6), and* E. avium* (5). Although there are studies assessing the impact of intensive animal rearing in the spread of VREs in the environment, there exist few reports of the contribution of feedlots to the dissemination of VREs. However, Bekele and Ashenafi [29] also screened* E. faecalis*,* E. faecium*, and* E. durans* from cattle faecal samples in Ethiopia. Faeces of animal origin are the primary source of these enterococcal isolates [30] and this explains our findings. In fact, Tanhi [31] also screened the same species in addition to* E. hirae* in cattle dung from farms in the Amathole district of South Africa. Our study differs with the above-mentioned reports in the sense that* E. hirae* was not detected in our samples but* E. casseliflavus*,* E. gallinarum, E. mundtii*, and* E. avium* were screened.* E. durans, E. casseliflavus*, and* E. mundtii* are environmental enterococci that are associated with plants and they are very common in herbivores faecal samples as a result of gut colonisation [32].* E. avium *is associated with bird droppings [32] and its presence in the water samples from the drinking troughs might be as a result of contamination by the birds that drink from the same troughs.* E. gallinarum* is predominant in chicken faeces but its presence in cattle and pig faecal samples has also been demonstrated [32, 33]. Enterococci were present in the feedlot soil samples as a result of contamination by the cattle faeces [32]. In fact, the soil from these feedlots was used as manure in the neighbouring farms.* E. durans* was the most predominant enterococcal isolate screened in this survey, followed by* E. faecium*,* E. gallinarum*, and* E. faecalis*.

The most-encountered vancomycin resistance gene in this study was* vanA* (62%), followed by* vanC* (21%) and* vanB* (17%). The highest number of VREs was* E. durans* strains and they mostly possessed* vanA* resistance gene (84 VR* E. durans*). The VR* E. mundtii* and VR* E. avium* isolated possessed only* vanC *resistance gene; meanwhile the other vancomycin resistant enterococci species isolated possessed* vanA* and* vanB* resistance genes in addition to* vanC* gene. Several studies worldwide established a link between vancomycin resistance and the usage of avoparcin (a glycopeptide analogue of vancomycin) as a growth promoter in animal husbandry [2, 5, 8, 9, 34, 35]. Although enterococci demonstrate intrinsic resistance to a large number of antibiotics (penicillins, cephalosporins, monobactams...etc.) [36], the findings of this study constitute a source of concern because avoparcin has been banned worldwide since 1997 [35]; there is therefore a need to further investigate the possible relationship between the antimicrobials used in these feedlots and the emergence of vancomycin resistance in the enterococcal isolates. Neither the feedlots owners nor their veterinaries disclosed data and information about the antimicrobials used in the investigated feedlots. Moreover, the unavailability or scarcity of data from wholesale suppliers and the Department of Agriculture, Forestry, and Fisheries of South Africa makes it difficult to establish a link between antimicrobial usage in the feedlots and the observations made in this study. VREs were also detected in faecal samples of animal origin in other areas of South Africa [31, 36] and in other parts of the world [8–11, 30, 33, 37–39] and these studies were consistent with our findings.

Tetracycline efflux pump genes were also detected in 138 (78%) VRE isolates.* msrA/B* was the tetracycline resistance gene mostly detected among the tetracycline resistant VREs (63%), followed by the* tetL* gene (32%).* tetK* and* mefA* were detected only in 15% and 5% of the tetracycline resistant VREs. Some VREs possessed more than one tetracycline resistance gene (31%) and the most encountered tetracycline resistance gene pattern was* tetL-msrA/B*. Several studies have linked the detection of tetracycline resistance genes in enterococci to the usage of tetracyclines in intensive animal rearing as growth promoters or therapeutic regiments [17]. Multidrug resistance in VREs is well documented [37]. 98% of the VRE isolates screened in this investigation were resistant to vancomycin and linezolid which were the drugs of choice for the treatment of enterococcal infections until the emergence of VREs worldwide. This finding is consistent with other reports worldwide [2, 4, 26, 30, 31, 33, 36–39] although the proportions recovered varied from one study to another because of the samples source and the samples size. Moreover, the majority of the isolates in this study were also resistant to penicillin and erythromycin (94% and 82%, resp.); meanwhile only 13% of the isolates were resistant to chloramphenicol and no resistance to ciprofloxacin was recorded. Nonnegligible proportions of the isolates were also resistant to tetracycline (64%), ampicillin (47%), and amoxicillin (40%). Antimicrobial resistance has always been linked to antimicrobial usage [9]. Although we had no access to data on antimicrobial usage in the investigated feedlots, these multidrug resistant isolates might have emerged as a result of antimicrobials usage for either prophylactic (added into animal feeds) or therapeutic measures or furthermore as a result of their use as growth promoters. In fact, the analysis of the clusters generated with the inhibition zone diameters proves that the isolates in this study were exposed to the same antibiotics in the different feedlots. Resistance to vancomycin is attributed to the possession of vancomycin resistance genes [10]; this explains our findings in the sense that antimicrobial susceptibility testing was carried out only on VRE isolates. Even though we reported a few resistant isolates to chloramphenicol and no resistance to ciprofloxacin, several studies reported high enterococcal resistance to ciprofloxacin and chloramphenicol in pig farms [31, 33, 34, 36] and in poultry [39]. It was associated with the possession of resistance genes that resulted from the therapeutic and prophylactic usage of advocin and chloramphenicol in animals. Furthermore, it was reported that resistance to tetracycline, ampicillin, amoxicillin, penicillin, and erythromycin is associated with the possession of resistance genes to these antibiotics, as a result of the widespread use of chlortetracycline, amoxicillin, penicillin, and erythromycin in intensive animal rearing either for disease control or as feed supplements or growth promoters [33, 40]. Coselection of resistance genes located on the same mobile genetic elements does occur as a result of the usage of different antibiotics in animal rearing [11–16]. Moreover, manure and soil or water contaminated with animals excreta are hotspots of isolates carrying mobile genetic resistance elements that can be transferred horizontally and vertically to animals/humans commensals and pathogens, which will find their way through previously described mechanisms, into the environment and the food chain [35, 41, 42]. Nevertheless, there is a need to further investigate the possession of other antibiotic resistance genes (such as* ermB*,* strA, pbp5, *and* gyr*) by the VREs screened in this study in order to further understand their resistance patterns.

Out of the 176 VREs screened in this study, 86 (49%) possessed virulence genes, namely,* gelE*,* asa1*,* hyl*,* cylA*, and* esp* genes. The virulent isolates displayed a variety of virulence patterns (Table 5) but the* gelE-hyl* virulence pattern mostly occurred (30 isolates) and was detected in most VR* E. durans* isolates.* gelE* and* esp* virulence genes were also detected in VRE isolates from previous studies [31, 36, 43] but not* cylA*,* asa1*, and* hyl* genes, as compared to our findings. However, not all isolates that possess the* gelE* gene do express gelatinase or *β*-haemolysis activity [18, 44], which constitute important attributes in enterococcal pathogenesis. Nevertheless, this fact does not mean that the virulent VREs isolated in this study are not pathogenic even though phenotypic virulence assays should be conducted to determine if all the amplified virulence genes are expressed by the isolates.* cylA* is one of the genes that code for the production of cytolysin, a protein which enables pathogenic enterococci to escape the host immune system by destroying macrophages and neutrophils [18]. It was not detected in the VR* E. casseliflavus*, VR* E. mundtii*, and VR* E. avium* isolates screened in this study, but it was amplified in VR* E. durans* (5), VR* E. faecalis* (1), VR* E. faecium* (1), and VR* E. gallinarum* (1) isolates. Other studies have reported the presence of* cylA* virulence gene in* E. faecium* and* E. faecalis* [18]. The detection of virulence genes in these isolates is a source of concern because of the health implications that could arise from their dissemination into the different ecological niches.

## 5. Conclusion

Potentially pathogenic vancomycin resistant enterococci were detected in samples from the 6 feedlots of the North West province in South Africa. The results reported in this investigation shed more light upon the impact of extensive usage of antimicrobials in intensive animal rearing and its implications on public health. Antimicrobial resistance and virulence genes are genetic mobile elements that can be transmitted horizontally and vertically to commensals and pathogens of warm blooded animals. Through well-understood mechanisms, this can lead to the spread of potentially pathogenic resistant strains into the environment and consequently into the food chain. Reports of multidrug resistant clinical and environmental isolates from community patients are quite unsettling because of the challenge that finding an appropriate therapeutic regime in such cases represents, not only for medical practitioners but also for researchers. Life-threatening conditions and diseases such as HIV-AIDS and diabetes motivate the urging need to address issues on the extensive usage of antimicrobials in intensive animal rearing and farming because its health implications cannot be overemphasised.

## Figures and Tables

**Table 1:** Sample types of this study.

District	Sampling area	Faecal samples	Drinking troughs water samples	Soil samples from the Kraals
Bojanala Platinum District	Swartfontein	147	4	4
Ngaka Modiri Molema District	Mafikeng	61	4	4
	Zeerust	46	4	4
	Rooigron	52	4	4
	Koster	38	4	4
Dr. Kenneth Kaunda	Potchefstroom	40	4	4

Total	6	384	24	24

**Table 2:** Oligonucleotide primers used in this study.

Genes	Target/primer	Sequences (5′-3′)	Size (bp)	PCR conditions	Reference
16S rRNA gene	16S rRNA	F: TGCATTAGCTAGTTGGTG	356	Denaturation 95°C for 4 min, 30 cycles at 95°C 30s, 58°C 60s, 72°C 60s, and 72°C 10 min	[20]
		R: TTAAGAAACCGCCTGCGC			
Species specific genes	*E. faecalis*	F: CACCTGAAGAAACAGGC	475	Denaturation 95°C for 4 min, 30 cycles at 95°C for 30 s, 55°C 60s, 72°C 60s, and 72°C for 7 min	[21]
		R: ATGGCTACTTCAATTTCACG			
	*E. faecium*	F: GAGTAAATCACTGAACGA	1091		
		R: CGCTGATGGTATCGATTCAT			
	*E. durans*	F: TTATGTCCCWGTWTTGAAAAATCAA	295		[22]
		R: TGAATCATATTGGTATGCAGTCCG			
	*E. gallinarum*	F: GGTATCAAGGAAACCTC	173		
		R: CTTCCGCCATCATAGCT			
	*E. hirae*	F: CTTTCTGATATGGATGCTGTC	187		
		R: TAAATTCTTCCTTAAATGTTG			
	*E. mundtii*	F: CAGACATGGATGCTATTCCATCT	98	Denaturation 95°C for 4 min, 30 cycles at 95°C for 30 s, 60°C 60s, 72°C 60s, and 72°C for 7 min	
		R: GCCATGATTTTCCAGAAGAAT			
	*E. casseliflavus*	F: TCCTGAATTAGGTGAAAAAAC	288	Denaturation 95°C for 4 min, 30 cycles at 95°C for 30 s, 55°C 60s, 72°C 60s, and 72°C for 7 min	
		R: GCTAGTTTACCGTCTTTAACG			
	*E. avium*	F: GCTGCGATTGAAAAATATCCG	368	Denaturation 95°C for 4 min, 30 cycles at 95°C for 30 s, 55°C 60s, 72°C 60s, and 72°C for 7 min	
		R: AAGCCAATGATCGGTGTTTTT			

Vancomycin resistance genes	*vanA*	F: GGGAAAACGACAATTGC	732	Denaturation 94°C 3 min, 30 cycles at 94°C 60 s, 54°C 60 s, 72°C 60 s, and 72°C 10 min	[21]
		R: GTACAATGCGGCCGTTA			
	*vanB*	F: ACGGAATGGGAAGCCGA	647		
		R: TGCACCCGATTTCGTTC			
	*vanC1/2*	F: ATGGATTGGTAYTKGTAT	815/827		
		R: TAGCGGGAGTGMCYMGTAA			
Tetracycline resistance genes	*msrA/B*	F: GCAAATGGTGTAGGTAAGACAACT	400	Denaturation 95°C 180 s, 35 cycles at 93°C 30 s, 55°C 120 s, and 72°C 90 s	[13]
		R: ATCATGTGATGTAAACAAAAT			
	*mefA*	F: AGTATCATTAATCACTAGTGC	348		
		R: TTCTTCTGGTACTAAAAGTGG			
	*tetL*	F: ATAAATTGTTTCGGGTCGGTAT	1107		
		R: AACCAGCCAACTAATGACAATGAT			
	*tetK*	F: TATTTTGGCTTTGTATTCTTTCAT	1159	Denaturation 95°C 60s, 35 cycles at 50°C 60 s, 72°C 30 s, and 72°C 300 s	
		R: GCTATACCTGTTCCCTCTGATAA			
	*tetM*	F: GCAGAATATACCATTCACATCGAAGT	700	Denaturation 95°C for 60s, followed by 40 cycles of 95°C for 15s, 60°C for 60s, and 72°C for 60s	[17]
		R: AAACCAATGGAAGCCCAGAA			

Virulence genes	*asa1*	F: GCACGCTATTACGAACTATGA	375	Denaturation 95°C 3 min, 30 cycles at 95°C 30s, 56°C 30s, 72°C 60s, and 72°C 10 min	[23]
		R: TAAGAAAGAACATCACCACGA			
	*gelE*	F: TATGACAATGCTTTTTGGGAT	213		
		R: AGATGCACCCGAAATAATATA			
	*cylA*	F: ACTCGGGGATTGATAGGC	688		
		R: GCTGCTAAAGCTGCGCTT			
	*esp*	F: AGATTTCATCTTTGATTCTTGG	510		
		R: AATTGATTCTTTAGCATCTGG			
	*hyl*	F: ACAGAAGAGCTGCAGGAAATG	278		
		R: GACTGACGTCCAAGTTTCCAA			

**Table 3:** Distribution of enterococcal species per sampling site.

Isolates species	Sites						Total
	Mafikeng	Koster	Potchefstroom	Roigron	Zeerust	Swartfontein	
*E. faecalis*	4	6	7	4	2	3	26
*E. faecium*	3	13	4	5	4	1	30
*E. durans*	27	18	13	10	5	126	199
*E. gallinarum*	4	4	2	3	3	2	18
*E. casseliflavus*	0	2	0	2	0	1	5
*E. mundtii*	0	0	0	3	3	0	6
*E. avium*	1	0	1	2	1	0	5
*E. hirae*	0	0	0	0	0	0	0

Total identified isolates	39	43	27	29	18	133	289
Total unidentified	30	23	25	65	36	59	238
Total number isolates	69	66	52	94	54	192	527

**Table 4:** Predominant multidrug resistance patterns observed among the isolates.

Number of isolates	Multidrug resistance pattern	Sites (*∗*number of isolates)
10	VAN^R^-PEN^R^-LIN^R^-ERY^R^	Zwartfontein (7), Mafikeng (2), and Roigron (1)
21	TET^R^-AMP^R^-AMX^R^-VAN^R^-PEN^R^-LIN^R^-ERY^R^	Zwartfontein (5), Potchefstroom (8), Koster (2), Zeerust (4), and Roigron (2)
13	TET^R^-AMP^R^-AMX^R^-VAN^R^-CHL^R^-PEN^R^-LIN^R^-ERY^R^	Zwartfontein(4), Mafikeng (2), Roigron (1), Potchefstroom (2), Koster (1), and Zeerust (3)
18	AMP^R^-AMX^R^-VAN^R^-PEN^R^-LIN^R^-ERY^R^	Zwartfontein (9), Zeerust (2), Roigron (4), and Koster (3)
17	TET^R^-AMP^R^-VAN^R^-PEN^R^-LIN^R^-ERY^R^	Zwartfontein (1), Mafikeng (4), Koster (5), Zeerust (3), Potchefstroom (4), and Roigron (1)
14	TET^R^-VAN^R^-PEN^R^-LIN^R^-ERY^R^	Zwatfontein (2), Mafikeng (6), and Koster (6)

VAN=vancomycin; TET=tetracycline; AMP=ampicillin; AMX=amoxicillin; ERY=erythromycin; LIN=linezolid; CHL=chloramphenicol; PEN=penicillin.

**Table 5:** Virulence genes patterns in the VRE isolates from the different sampling sites.

Species	Virulence factors	Number of positive isolates	Sites (**∗**number of isolates)
*E. faecalis*	*gelE*	4	Mafikeng (1), Potchefstroom (2), and Swartfontein (1)
	*hyl*	1	Roigron (1)
	*cylA-hyl*	1	Roigron (1)
	*gelE-hyl*	2	Zeerust (1), Roigron (1)
*E. faecium*	*asa1*	1	Roigron (1)
	*hyl*	1	Potchefstroom (1)
	*gelE*	1	Swartfontein (1)
	*gelE-cylA*	1	Koster (1)
	*gelE-hyl*	3	Koster (2), Swartfontein (1)
	*asa1-gelE-esp-hyl*	1	Mafikeng (1)
*E. durans*	*asa1*	2	Swartfontein (2)
	*cylA*	1	Koster (1)
	*gelE*	8	Mafikeng (2), Potchefstroom (2), Swartfontein (3), and Roigron (1)
	*hyl*	2	Swartfontein (2)
	*asa1-gelE*	4	Mafikeng (2), Swartfontein (1), and Roigron (1)
	*gelE-hyl*	21	Koster (1), Zeerust (1), and Swartfontein (19)
	*asa1-hyl*	5	Mafikeng (1), Swartfontein (4)
	*cylA-esp*	1	Swartfontein (1)
	*esp-hyl*	1	Swartfontein (1)
	*gelE-cylA*	2	Koster (2)
	*asa1-esp-hyl*	2	Swartfontein (2)
	*asa1-gelE-hyl*	6	Swartfontein (6)
	*gelE-esp-hyl*	1	Koster (1)
	*asa1-gelE-esp-cylA*	1	Mafikeng (1)
*E. gallinarum*	*hyl*	2	Swartfontein (1), Zeerust (1)
	*asa1-gelE*	1	Mafikeng (1)
	*gelE-hyl*	4	Koster (1), Roigron (3)
	*asa1-cylA-hyl*	1	Swartfontein (1)
	*gelE-esp-hyl*	1	Mafikeng (1)
*E. casseliflavus*	*asa1*	1	Swartfontein (1)
	*gelE*	2	Roigron (2)
*E. mundtii*	None	0	- - - -
*E. avium*	*gelE*	1	Zeerust

Total	86		

**Table 6:** Cluster distribution of the isolates.

Sampling site	Sample type	Cluster 1A	Cluster 1B	Cluster 2
		N = 69	N = 2	N = 1
Swartfontein	Faecal	28 (40%)	0 (0%)	0 (0%)
	Soil	2 (3%)	0 (0%)	0 (0%)
	Water	1 (1%)	0 (0%)	0 (0%)
Mafikeng	Faecal	8 (12%)	1 (50%)	0 (0%)
	Soil	0 (0%)	0 (0%)	0 (0%)
	Water	1 (1%)	0 (0%)	0 (0%)
Zeerust	Faecal	2 (3%)	0 (0%)	0 (0%)
	Soil	0 (0%)	0 (0%)	0 (0%)
	water	2 (3%)	0 (0%)	0 (0%)
Roigron	Faecal	8 (12%)	0 (0%)	0 (0%)
	Soil	0 (0%)	1 (50%)	0 (0%)
	Water	2 (3%)	0 (0%)	0 (0%)
Koster	Faecal	8 (12%)	0 (0%)	1 (100%)
	Soil	0 (0%)	0 (0%)	0 (0%)
	Water	1 (1%)	0 (0%)	0 (0%)
Potchefstroom	Faecal	3 (4%)	0 (0%)	0 (0%)
	Soil	3 (4%)	0 (0%)	0 (0%)
	Water	0 (0%)	0 (0%)	0 (0%)

## Data Availability

Representative bacterial 16S rRNA sequences were submitted and are accessible at the GenBank database under accession numbers MK086096- MK086108. Moreover, the data used to support the findings of this study are available from the corresponding author upon request.

## Abbreviations

VREs: Vancomycin-resistant enterococci
VRE: Vancomycin-resistant* Enterococcus*
*esp*: Enterococcal surface protein
*hyl*: Hyaluronidase
*cyl*: Cytolysin
*gelE*: Gelatinase
HIV: Human immunodeficiency virus
AIDS: Acquired immunodeficiency syndrome
PCR: Polymerase chain reaction
DNA: Deoxyribonucleic acid
rRNA: Ribosomal ribonucleic acid.

## Additional Points

*Study Limitations.* There was no access to data on the antimicrobials used in the investigated feedlots, unavailability, or scarcity of data from wholesale suppliers and the Department of Agriculture, Forestry, and Fisheries of South Africa.

## References

[1] Domig K. J., Mayer H. K., Kneifel W. (2003). Methods used for the isolation, enumeration, characterisation and identification of *Enterococcus spp.* media for isolation and enumeration. *International Journal of Food Microbiology*.

[2] Mannu L., Paba A., Daga E. (2003). Comparison of the incidence of virulence determinants and antibiotic resistance between *Enterococcus faecium* strains of dairy, animal and clinical origin. *International Journal of Food Microbiology*.

[3] Morrison D., Woodford N., Barrett S. P., Sisson P., Cookson B. D. (1999). DNA banding pattern polymorphism in vancomycin-resistant Enterococcus faecium and criteria for defining strains. *Journal of Clinical Microbiology*.

[4] Sharifi Y., Hasani A., Ghotaslou R., Naghili B., Aghazadeh M., Milani M. (2013). Virulence and antimicrobial resistance in enterococci isolated from urinary tract infections. *Advanced Pharmaceutical Bulletin*.

[5] Myllys V., Rautala H. (1995). Characterization of clinical mastitis in primiparous heifers. *Journal of Dairy Science*.

[6] Simner P. J., Adam H., Baxter M. (2015). Epidemiology of vancomycin-resistant enterococci in Canadian hospitals (CANWARD study, 2007 to 2013). *Antimicrobial Agents and Chemotherapy*.

[7] Noble W. C., Virani Z., Cree R. G. A. (1992). Co-transfer of vancomycin and other resistance genes from *Enterococcus faecalis* NCTC 12201 to *Staphylococcus aureus*. *FEMS Microbiology Letters*.

[8] Bager F., Madsen M., Christensen J., Aarestrup F. M. (1999). Avoparcin used as a growth promoter is associated with the occurrence of vancomycin-resistant *Enterococcus faecium* on Danish poultry and pig farms. *Preventive Veterinary Medicine*.

[9] Marshall B. M., Levy S. B. (2011). Food animals and antimicrobials: impacts on human health. *Clinical Microbiology Reviews*.

[10] LeClercq R., Derlot E., Duval J., Courvalin P. (1988). Plasmid-mediated resistance to vancomycin and teicoplanin in *Enterococcus faecium*. *The New England Journal of Medicine*.

[11] Seo K. S., Lim J. Y., Yoo H. S., Bae W. K., Park Y. H. (2005). Comparison of vancomycin-resistant enterococci isolates from human, poultry and pigs in Korea. *Veterinary Microbiology*.

[12] Tansuphasiri U., Khaminthakul D., Pandii W. (2006). Antibiotic resistance of enterococci isolated from frozen foods and environmental water. *Southeast Asian Journal of Tropical Medicine and Public Health*.

[13] Molale L. G., Bezuidenhout C. C. (2016). Antibiotic resistance, efflux pump genes and virulence determinants in *Enterococcus spp.* from surface water systems. *Environmental Science and Pollution Research*.

[14] Collins N. A., Merapelo D. M. (2011). Detection of *Enterococcus species* in groundwater from some rural communities in the Mmabatho area, South Africa: a risk analysis. *African Journal of Microbiology Research*.

[15] Nateghian A., Fallah F., Daghighi Z., Goudarzi H., Hashemi A., Robinson J. L. (2016). Detection of virulence genes in resistant enterococci isolated from pediatric patients at high risk for nosocomial infections. *Diagnostic Microbiology and Infectious Disease*.

[16] Ateba C. N., Mohapi M. I. (2013). Isolation of vancomycin resistant enterococci isolated from leafy vegetables (lettuce) from North West Province. *Life Science Journal*.

[17] Wilcks A., Andersen S. R., Licht T. R. (2005). Characterization of transferable tetracycline resistance genes in *Enterococcus faecalis* isolated from raw food. *FEMS Microbiology Letters*.

[18] Upadhyaya G. P. M., Ravikumar K. L., Umapathy B. L. (2009). Review of virulence factors of *Enterococcus*: an emerging nosocomial pathogen. *Indian Journal of Medical Microbiology*.

[19] O'Brien T. F. (2002). Emergence, spread, and environmental effect of antimicrobial resistance: how use of an antimicrobial anywhere can increase resistance to any antimicrobial anywhere else. *Clinical Infectious Diseases*.

[20] Kariyama R., Mitsuhata R., Chow J. W., Clewell D. B., Kumon H. (2000). Simple and reliable multiplex PCR assay for surveillance isolates of vancomycin-resistant enterococci. *Journal of Clinical Microbiology*.

[21] Depardieu F., Perichon B., Courvalin P. (2004). Detection of the van alphabet and identification of enterococci and staphylococci at the species level by multiplex PCR. *Journal of Clinical Microbiology*.

[22] Jackson C. R., Fedorka-Cray P. J., Barrett J. B. (2004). Use of a genus- and species-specific multiplex PCR for identification of enterococci. *Journal of Clinical Microbiology*.

[23] Vankerckhoven V., Van Autgaerden T., Vael C. (2004). Development of a multiplex PCR for the detection of *asa1, gelE, cylA, esp*, and *hyl* genes in enterococci and survey for virulence determinants among european hospital isolates of *Enterococcus faecium*. *Journal of Clinical Microbiology*.

[24] Bauer A. W., Kirby W. M., Sherris J. C., Turck M. (1966). Antibiotic susceptibility testing by a standardized single disk method. *American Journal of Clinical Pathology*.

[25] Arias C. A., Murray B. E., Mandell G. L., Bennett J. E., Dolin R. (2009). *Enterococcus species*, *Streptococcus bovis* group and *Leuconostoc species*. *Mandell, Douglas, and Bennett's Principles and Practice of Infectious Diseases*.

[26] Larsen J., Schønheyder H. C., Lester C. H., Olsen S. S., Porsbo L. J., Garcia-Migura L. (2010). Porcine-origin gentamicin-resistant *Enterococcus faecalis* in humans, Denmark. *Emerging Infectious Diseases*.

[27] Fisher K., Phillips C. (2009). The ecology, epidemiology and virulence of *Enterococcus*. *Microbiology*.

[28] Courvalin P. (2005). Genetics of glycopeptide resistance in Gram-positive pathogens. *International Journal of Medical Microbiology*.

[29] Bekele B., Ashenafi M. (2010). Distribution of drug resistance among enterococci and Salmonella from poultry and cattle in Ethiopia. *Tropical Animal Health and Production*.

[30] Li P., Wu D., Liu K. (2014). Investigation of antimicrobial resistance in *Escherichia coli* and Enterococci isolated from tibetan pigs. *PLoS One*.

[31] Tanih G. N. (2016). *Genotypic and Phenotypic characterization of enterococci from cow dung and environmental water sources in three selected dairy farms in Amathole District*.

[32] Salminen S., Wright A. V., Ouwehand A. (2004). *Lactic Acid Bacteria*.

[33] Pruksakorn C., Pimarn C., Boonsoongnern A., Narongsak W. (2016). Detection and phenotypic characterization of vancomycin-resistant enterococci in pigs in Thailand. *Agriculture and Natural Resources*.

[34] Aarestrup F. M., Kruse H., Tast E., Hammerum A. M., Jensen L. B. (2000). Associations between the use of antimicrobial agents for growth promotion and the occurrence of resistance among *Enterococcus faecium* from broilers and pigs in Denmark, Finland, and Norway. *Microbial Drug Resistance*.

[35] Tatsing Foka F. E., Kumar A., Ateba C. N. (2018). Emergence of vancomycin-resistant enterococci in South Africa: implications for public health. *South African Journal of Science*.

[36] Iweriebor B. C., Obi L. C., Okoh A. I. (2015). Virulence and antimicrobial resistance factors of Enterococcusspp. isolated from fecal samples from piggery farms in Eastern Cape, South Africa Ecological and evolutionary microbiology. *BMC Microbiology*.

[37] Garcia-Migura L., Pleydell E., Barnes S., Davies R. H., Liebana E. (2005). Characterization of vancomycin-resistant *Enterococcus faecium* isolates from broiler poultry and pig farms in England and Wales. *Journal of Clinical Microbiology*.

[38] Peters J., Mac K., Wichmann-Schauer H., Klein G., Ellerbroek L. (2003). Species distribution and antibiotic resistance patterns of enterococci isolated from food of animal origin in Germany. *International Journal of Food Microbiology*.

[39] Ünal N., Aşkar Ş., Yildirim M. (2017). Antibiotic resistance profile of *Enterococcus faecium* and Enterococcus faecalis isolated from broiler cloacal samples. *Turkish Journal of Veterinary & Animal Sciences*.

[40] Moyane J., Jideani A., Aiyegoro O. (2013). Antibiotics usage in food-producing animals in South Africa and impact on human: Antibiotic resistance. *African Journal of Microbiology Research*.

[41] Ding G.-C., Radl V., Schloter-Hai B. (2014). Dynamics of soil bacterial communities in response to repeated application of manure containing sulfadiazine. *PLoS ONE*.

[42] Thanner S., Drissner D., Walsh F. (2016). Antimicrobial resistance in agriculture. *mBio*.

[43] Medeiros A. W., Pereira R. I., Oliveira D. V. (2014). Molecular detection of virulence factors among food and clinical *Enterococcus faecalis* strains in South Brazil. *Brazilian Journal of Microbiology*.

[44] Lauková A., Strompfová V., Kandričáková A. (2015). Virulence factors genes in enterococci isolated from beavers (Castor fiber). *Folia Microbiologica*.

